# On the Pulvis Antimonialis of the London Pharmacopœia

**Published:** 1816-05

**Authors:** George Pearson

**Affiliations:** George Street, Hanover Square.


THE LONDON

### Medical and Physical Journal.

5 OF VOL. XXXV.] MAY, 1816. [NO. 207.

"For many fortunate discoveries in medicine, and for the detection of numerous errors, the world is indebted to the rapid circulation of Monthly Journals; and there never existed any work to which the Faculty in EUROPE and AMERICA were under deeper obligations than to the Medical and Physical Journal of London, now forming a long, but an invaluable, series."—RUSH.

### *For the London Medical and Physical Journal.*

### *On the Pulvis Antimonialis of the London Pharmacopœia;* by GEORGE PEARSON, M.D. *of George Street, Hanover Square.*

ALTHOUGH I have not thought proper to take any part in the composition of the London Pharmacopœias, I should not, perhaps, be altogether justifiable in withholding a few remarks on the article *pulvis antimonialis*, in consequence of your reference to my analysis, published in the Philosophical Transactions of 1791. You, as editors too, are well entitled to attention for your judicious and able criticisms of the whole work; of which I hope, for the sake of humanity, the College will avail themselves in a future edition.

As you rightly observe, (Journal, Dec. 1815, p. 488,) "it seems very extraordinary conduct in the translator, to pass unnoticed the alteration in the prescription of the article in question, while he deems it important to fill a page with a quotation of the absurd deception from the records of Chancery;" thereby attaching importance, and showing respect, for what has long been considered by the public in general in the light you describe. Further, this proceedure, if really one can find it at all reasonable, must arise from a belief that the specification contains the prescription for the *James's powder*, as sold to the public for the last forty or fifty years; and as, according to the analysis which I have above mentioned, the former *pulvis antimonialis* and the James's powder are identical, the inference must be, that the medical public ought not to confide any longer in the use of the Pharmacopœia prescriptions in place of the *nostrum*. If the translator thought proper to write his strictures on the point of the James's powder, as stated in the specification, surely the public had a right to expect that he should have given evidence against the evidence I have published, and which has been confirmed subsequently by Mr. Chenevix, that the two articles are identical, otherwise he has only opposed the specification, of the truth of which there is no proof, to the published proofs by other persons.

But, whether the two named substances be identical or not, as you justly remark, why has the translator left us in the dark as to the views of the Committee of the College, in reducing the proportion of the antimony to the bone shaving to one half? The present prescription of the *pulvis antimonialis*, has no authority but the committee; the former had not only testimonies that it was identical with James's powder, but also, as I have published in the Philosophical Transactions for 1791, it is the prescription of Schröder in 1640, or thereabouts, and also of Lile. There is much collateral evidence that Baron Schwanberg introduced this medicine into England, and that it was not his invention, having been published above sixty years before his time by Schröder. Hence, then, the medical public find an established medicine of great importance altered most materially in its composition without any assigned reason. Further, it is with much justice feared that many disappointments in practice have been experienced in the use of the new prescription, owing to the dose not being varied from that of the formula in the former editions of the Pharmacopœia.

*March* 20, 1816.

---

*For the London Medical and Physical Journal.*

